# Objektive Bewertung peripherer Vestibulopathien als Beitrag zur wissenschaftlich fundierten HNO-Begutachtung

**DOI:** 10.1007/s00106-025-01643-y

**Published:** 2025-07-10

**Authors:** Leif Erik Walther

**Affiliations:** HNO-Gemeinschaftspraxis, Main-Taunus-Zentrum, 65843 Sulzbach (Taunus), Deutschland

**Keywords:** Vestibuläre Funktionsuntersuchungen, Schwindel, Gleichgewichtsstörungen, M. Menière, Kopfimpulstest, Vestibular function tests, Vertigo, Dizziness, Ménière’s disease, Head impulse test

## Abstract

Der wissenschaftliche Fortschritt in der Therapie von Schwindelsyndromen hat zu einem Paradigmenwechsel in der diagnostischen Herangehensweise, einem einheitlichen Vokabular und international standardisierten Kriterien geführt. Objektive Methoden einschließlich vestibulär evozierter myogener Potenziale und des Video-Kopfimpulstests, haben sich in der HNO-Begutachtung durchgesetzt. Auf dieser Grundlage sind neue Bewertungskriterien auf objektiver Grundlage entwickelt worden, die dem Auftraggeber im Rechtskontext mehr Klarheit verschaffen als die bisherige Bewertung mit vorwiegend subjektiven Maßstäben und damit plausibel und nachvollziehbar sind. Im Rahmen der finalen und kausalen Begutachtung ist es jetzt möglich, eine im Vollbeweis gesicherte Gesundheitsstörung im HNO-Fachbereich auf der Grundlage objektiver Kriterien einzuschätzen.

## Abstract (en)

Scientific progress in the diagnosis and treatment of dizziness syndromes has led to a paradigm shift in diagnostic approaches, establishment of a unified international vocabulary, and standardized criteria. Objective methods, including vestibular evoked myogenic potentials and the video head impulse test, are now widely used in otorhinolaryngologic assessments. According to these advancements, new evaluation criteria based on objective measures have been developed. These provide greater clarity in the legal context compared to previous evaluations which were based primarily on subjective standards, thus making the assessments more plausible and comprehensible. It is now possible to definitively substantiate a defined health disorder or functional impairment in the field of otorhinolaryngology with full evidence based on objective criteria.

Im HNO-Fachbereich wurde in den letzten Jahren eine Neubewertung im Rahmen der Begutachtung von Erkrankungen mit dem Leitsymptom „Schwindel“ vorgenommen [1–5].

Periphere Vestibulopathien können heute einer gesicherten Gesundheitsstörung zugeordnet werden

Zum einen hat sich gezeigt, dass krankheitsspezifische Bewertungsmaßstäbe für periphere Vestibulopathien mit dauerhaften Beschwerden und solche mit Anfallscharakter auf der Grundlage objektiver Kriterien und Qualitätsstandards für die Diagnostik notwendig sind (z. B. benigner paroxysmaler Lagerungsschwindel [BPLS], M. Menière, bilaterale Vestibulopathie). Zum anderen wurde deutlich, dass die Bewertung HNO-spezifischer Krankheitsbilder (peripherer Vestibulopathien) mit den bisherigen Tabellen für „Schwindel“ bzw. „vestibuläre Störungen“ mit dem Maßstab des wissenschaftlichen Erkenntnisstands nicht mehr dem wissenschaftlichen Fortschritt entsprechen, eine Dominanz subjektiver Bewertungskriterien besteht und die Fachgrenzen nicht eindeutig definiert sind [6–8]. Bei der Beurteilung HNO-spezifischer peripherer Vestibulopathien lässt sich heute auf der Grundlage objektiver Messergebnisse mittels quantitativer, seitengetrennter und sensorspezifischer Analyse eine Zuordnung zu einer gesicherten Gesundheitsstörung mit operationalisierter Diagnose (z. B. nach ICD-Klassifikation, International Statistical Classification of Diseases and Related Health Problems) durchführen, die dem Auftraggeber auf einer objektiven Grundlage Klarheit verschafft [9, 10].

## Grundlagen der HNO-Begutachtung bei „Schwindel“

Das HNO-Fachbereich beschäftigt sich vordergründig mit peripheren Vestibulopathien, Erkrankungen des Gleichgewichtsorgans und des Gleichgewichtsnervs bis etwa zum Eintritt in den Hirnstamm. Hinzu kommen fachübergreifende Kenntnisse über Schwindelsyndrome, eine grobe Einschätzung zentraler vestibulärer Funktionsstörungen aus dem Fachgebiet der Neurologie und die Kenntnis über Zusammenhänge mit anderen Fachgebieten (Innere Medizin, Orthopädie, Psychiatrie, Psychosomatik usw.). In Analogie zum Tinnitus können durch den HNO-Gutachter heute auch funktionelle Schwindelsyndrome integrierend eingeschätzt und bewertet werden, wenn diese nicht den Hauptanteil der Diagnose darstellen. Die Fachkompetenz regelt die jeweils gültige Musterweiterbildungsordnung der Bundesärztekammer, die die fachlichen Inhalte des HNO-Arztes für das Symptom „Schwindel“ festlegt [11].

### Internationales Vokabular und Krankheitsdefinitionen

Die Internationale Gesellschaft für Neurootologie, Vestibularismedizin und -forschung (Bàràny Society) veröffentlicht seit 2009 diagnostische Kriterien für verschiedene Schwindelsyndrome [12].

Mit dem Standardisierungsprozess der Bàràny-Gesellschaft für Neurootologie hat sich ein fachübergreifendes Vokabular durchgesetzt

Mit dem Standardisierungsprozess hat sich ein fachübergreifendes Vokabular durchgesetzt, welches inzwischen auch international als „Standard“ im Rahmen der Diagnostik und Therapie von Schwindelsyndromen genutzt wird (s. auch die interdisziplinäre S2k-Leitlinie „Vestibuläre Funktionsstörungen“) [13]. Diese einheitliche Fachterminologie bildet die Grundlage des wissenschaftlichen Fachvokabulars in HNO-Fachgutachten.

### Ermittlung der Gesundheitsstörung im Vollbeweis

Voraussetzung für eine Bewertung der Auswirkungen im täglichen Leben und im Beruf bzw. eine Funktionsstörung im Rechtskontext beim subjektiven Symptom „Schwindel“ ist das Vorliegen einer Gesundheitsstörung im HNO-Fachbereich. Diese muss im Vollbeweis gesichert sein, objektiv nachgewiesen sein und mit einer Diagnose entsprechend einer internationalen Krankheitsklassifikation (z. B. ICD 10) benannt werden (z. B. Inkomplette periphere Vestibulopathie rechts, ICD 10: H 81.2). Diese Forderung besteht in jedem Rechts- und Versicherungsgebiet. Das Vorhandensein von „Schwindel“ (ICD 10 R.42) als Symptom ist nicht ausreichend für eine medizinische Bewertung. Transparenz und Gewissheit über eine fachspezifische Diagnose ergibt eine Differenzialdiagnostik mit objektiven Methoden im Rahmen einer gutachterlichen HNO-Untersuchung [3, 4, 9, 10, 14–16].

### Objektive vs. subjektive Bewertung

Gesetzgeber und Auftraggeber fordern im Rahmen der Begutachtung von medizinischen Sachverhalten eine objektive Herangehensweise an die Fragestellungen in den Beweisfragen auf der Basis des aktuellen wissenschaftlichen Erkenntnisstands zur Vorbereitung juristischer Entscheidungen in verschiedenen Rechts- und Versicherungsgebieten. Eine objektive Betrachtung basiert auf augenscheinlichen Fakten und Beweisen auf der Basis nachprüfbarer Dokumentationen (in den Akten) und messbarer Daten (im Rahmen der Untersuchung). Objektive Bewertungen sind nachvollziehbar und plausibel. Sie erleichtern eine Entscheidungsfindung. Subjektive Bewertungen sind hingegen von der persönlichen Wahrnehmung abhängig, sie variieren, weisen eine hohe Schwankungsbreite auf und lassen sich nicht augenscheinlich und messbar nachvollziehen [3, 4, 14–16].

## Methodik und Referenzbereiche objektiver vestibulärer Funktionsprüfungen

### HNO-Lehrmeinung

Periphere Vestibulopathien können heute präzise mit objektiven Methoden quantitativ, seiten- und sensorspezifisch diagnostiziert werden. Standardmethoden in der Diagnostik sind der Video-Kopfimpulstest für alle 3 Bogengänge (hochfrequenter Anteil des vestibulookulären Reflexes, VOR), die zervikalen und okulären vestibulär evozierten myogenen Potenziale (cVEMP, oVEMP) für die objektive Messung der überwiegenden Utrikulus- und Sakkulusfunktion in Luftleitung (500 Hz, Burstreizung) und die thermische Gleichgewichtsprüfung („niederfrequenter“ horizontaler VOR, hVOR).

Der HNO-Gutachter kann sich auf Basis objektiver Untersuchungsergebnisse auf eine Störung beziehen

Damit kann sich der HNO-Gutachter auf der Basis objektiver Untersuchungsergebnisse [17] und anhand neuer Diagnosekriterien (z. B. Bàràny-Konsensus-Dokumente, [13]) auf eine Störung (Gesundheitsschaden) in seinem Fachgebiet beziehen und diese eindeutig einer international klassifizierten Diagnose nach ICD zuordnen [9, 10]. Diese diagnostische Präzision und Objektivität erleichtern auch dem Auftraggeber die Wahrheitsfindung im Rechtskontext deutlich, da die Gesamteinschätzung der Auswirkungen der Funktionsstörung nicht mehr, wie bisher, überwiegend auf subjektiven Bewertungen und Testmethoden basiert (z. B. subjektiv geprägte Einschätzung der „Intensität“ und der „Belastung“) [6, 7].

### Mindeststandard und Referenzbereiche

Die objektiven Untersuchungsmethoden (z. B. Video-Kopfimpulstest für alle 3 Bogengänge, die zervikalen und okulären vestibulär evozierten myogenen Potenziale (cVEMP, oVEMP) in Luftleitung (500 Hz, Burstreizung) und die thermische Gleichgewichtsprüfung) werden gegenwärtig als Mindeststandard der apparativen Untersuchung im Rahmen einer HNO-Begutachtung bei „Schwindel“ empfohlen (Tab. 1). Bei peripheren Vestibulopathien mit Anfallscharakter sollte eine Objektivierung erfolgen (z. B. Dokumentation des Lagerungsnystagmus beim BPLS). Für die Ermittlung der vestibulären Kompensation wird die Erfassung von Spontan- und Kopfschüttelnystagmus (videookulographische Dokumentation, ggf. Videodatei) empfohlen. Die Befunde sollten mit den Befunden der Untersuchung der statischen (geringere Wertigkeit) und dynamische Komponente (Einsatz evaluierter Testverfahren) plausibel zusammenpassen. Mit dem objektiv messbaren Schädigungsgrad (*Ausmaß des Organschadens*) und dem *Grad der vestibulären Kompensation* stehen 2 unabhängige Parameter auf objektiver Basis zur Bewertung zur Verfügung, die nicht mehr nur auf subjektiven Beschwerden und Befunden (z. B. Intensität, Belastbarkeit) ermittelt werden [3, 4]. Referenzbereiche sollten mit der verwendeten Methodik in Analogie zur Labormedizin im Gutachten angegeben werden. Auch die Ergebnisse sollten (in Analogie zum Audiogramm) zur Verfügung gestellt werden, da diese eine Abweichung vom Normalbefund eindeutig belegen können. Das ermöglicht eine Einordnung und Nachvollziehbarkeit der Befunde.

**Tab. 1:** Methodik und Referenzbereiche objektiver Vestibularisprüfungen im Rahmen der HNO-Begutachtung

Testverfahren	Methodik	Referenzbereiche
Thermische Prüfung („niederfrequenter“ hVOR)	44°- und 30°-Wasserreizung des Gehörgangs, ggf. monothermal, Luft in Ausnahmefällen bei Trommelfelldefekten/Radikalhöhlen	Wasser: Seitendifferenz < 25 %, Luftreizung (keine quantitative Aussage, paradoxer Nystagmus [12])
Video-Kopfimpulstest („hochfrequenter“ VOR, 3 Bogengänge)	Heller Raum, Blickziel in 1,5 m, etwa 10–20 Kopfimpulse (15–20°), Dauer 150–200 ms, Geschwindigkeit 150–200°/s, 3 Bogengänge bds.	HVOR-Gain etwa ≤ 0,79, vertikale Bogengänge schwieriger zu prüfen, aber gut quantitativ zu beurteilen [12, 18]
Vestibulär evozierte myogene Potenziale (VEMP; überwiegende Sakkulus- und Utrikulusfunktion)	Zervikale (cVEMP) und okuläre Ableitungen (oVEMP) in Luftleitung, 500 Hz, Burst-Reizung 100 dB nHL, 1 ms Rise-Fall-Time, 2 ms Plateau, etwa 50–100 Mittelungen, cVEMP: EMG 50–250 µV, auch bei Ertaubung, bei Schallleitungsstörungen schwierig (Knochenleitungs-VEMP)	cVEMP: Latenz: 15,91 ms ± 8,28 ms (p13), 24,69 ms ± 12,80 ms (n23); Amplitude 113,05 µV ± 35,95 µV. AR: 13,83 % ± 10,87 % (< 43 %); oVEMP: Latenz: 11,35 ± 1 (n10); 16,3 ± 1,1 (p13), Amplitude: 7,70 ± 4,50 μV; AR: 17,07 % ± 12,90 %; < 36 % [19]

*AR* Amplitude Ratio (Amplituden-Verhältnis), *cVEMP* zervikale VEMP, *EMG *Elektromyographie,* hVOR* horizontaler VOR, *oVEMP* okuläre VEMP, *VEMP* vestibulär evozierte myogene Potenziale,* VOR* vestibulookulärer Reflex. *AR* Amplitude-Ratio (Amplituden-Verhältnis)

## HNO-Begutachtung

Bei allen gutachterlichen Fragestellungen mit dem Symptom „Schwindel“ sollte der HNO-Gutachter u. a. folgende klären:

- Liegt objektiv eine periphere Vestibulopathie/sensorische Funktionsstörung ohne und/oder mit Anfallscharakter im HNO-Fachgebiet vor? (Gesundheitsstörung nach ICD im Vollbeweis)?
- Wie ist diese charakterisiert (Lokalisation, Art, Ausmaß und Eigenschaften; Organschadensgrad; Tab. 2)?
- Wie ist das Ausmaß der vestibulären Kompensation (Kompensationsgrad, Tab. 3)?
- Welche Innenohrstörungen liegen noch vor?
- Lassen sich die Beschwerden/Auswirkungen mit der erforderlichen Wahrscheinlichkeit erklären oder gibt es Diskrepanzen (Begleiterkrankungen)? [4]

**Tab. 2:** Organschadentabelle für die Bestimmung des Schweregrads der Organschädigung (sensorische/neurale Störung) bei ein- oder beidseitigen peripheren vestibulären Störungen

Unwesentlich/kaum Grad 1	Leichtgradig Grad 2	Mittelgradig Grad 3	Schwergradig Grad 4
Z. B. kaum Reduktion der thermischen Erregbarkeit, etwa 25 bis < 30 % Seitendifferenz, kaum Reduktion des Gainwerts, kaum VEMP-Amplitudenreduktion	Thermische Erregbarkeit etwa 30–< 40 % Seitendifferenz, hVOR-Gain etwa > 0,6 oder Teilschäden der oberen Bogengänge einzeln oder kombiniert, unilateraler Teilschaden oder einseitige Vollschäden der Otolithenorgane, auch einzeln, keine offenen Rückstellsakkaden	Thermische Erregbarkeit etwa 40–< 90 % Seitendifferenz, hVOR-Gain ca. > 0,4–0,6, verdeckte Rückstellsakkaden und/oder offene Rückstellsakkaden, einseitiger Vollschaden beider oberer Bogengänge, bilateraler Schaden der Otolithenorgane, Schaden zweier korrespondierender oberer Bogengänge	Thermische Erregbarkeit etwa > 90 % Seitendifferenz, hVOR-Gain etwa < 0,4, offene Rückstellsakkaden, beidseitige Schädigung des hVOR-Gain (etwa < 0,6), Vollschaden auf einer Seite und bilaterale Vestibulopathie

*hVOR* horizontaler vestibulookulärer Reflex, *VEMP* vestibulär evozierte myogene Potenziale

**Tab. 3:** Kompensationstabelle zur Ermittlung des Grads der vestibulären Kompensation bei ein- oder beidseitigen peripheren vestibulären Störungen

Testverfahren/vestibuläre Kompensation	Schwach (Grad 1)	Mittelmäßig (Grad 2)	Stark (Grad 3)	Nahezu vollständig (Grad 4)
*Spontannystagmus*				
Video- oder Elektronystagmographie GLP* [°/s]	Starke bis vollständige Normabweichung (hochfrequent, etwa > 10°/s)	Mittelmäßige Normabweichung (mittelfrequent, etwa 5–10°/s)	Schwache Normabweichung (niederfrequent, etwa < 5°/s)	Ohne bis sehr schwache Normabweichung
*Kopfschüttelnystagmus*				
Nystagmusanzahl nach Provokation oder GLP* (°/s)	Starke bis vollständige Normabweichung	Mittelmäßige Normabweichung	Schwache Normabweichung	Ohne bis sehr schwache Normabweichung
	Etwa > 10–20 oder ca. > 15°/s	Etwa 5–10 oder etwa 5–15°/s	Etwa < 5 oder etwa < 5°/s	–
Statische Prüfungen**				
Ergebnis statischer Prüfungen**	Starke bis vollständige Normabweichung	Mittelmäßige Normabweichung	Schwache Normabweichung	Ohne bis sehr schwache Normabweichung
*Dynamische Prüfungen*				
Ergebnis dynamischer Prüfungen***	Starke bis vollständige Normabweichung	Mittelmäßige Normabweichung	Schwache Normabweichung	Ohne bis sehr schwache Normabweichung

*GLP: Geschwindigkeit der langsamen Nystagmusphase

**Geringere Wertigkeit als objektive Tests und dynamische Prüfungen

***Mehrere, möglichst komplexe evaluierte Testverfahren

### Eigenschaften peripherer Vestibulopathien ohne Anfallscharakter

Bei chronischen peripheren Vestibulopathien stehen dauerhafte Beschwerden aufgrund sensorischer Defizite und einem geringeren Grad der vestibulären Kompensation im Vordergrund. Teilschäden haben i. d. R. eine geringere Auswirkung als Vollschäden, und eine Beteiligung des horizontalen Bogengangs (hVOR) zeigt der Erfahrung nach schwergradigere Folgen (dominanter Bogengang) als die „untergeordneten“ vertikalen Bogengänge bzw. die Otolithenorgane. Beidseitige Störungen korrespondierender Bogengänge sind meist schwergradiger als einseitige (bilaterale Vestibulopathie). Objektiv nachweisbare (offene) Rückstellsakkaden im Video-Kopfimpulstest (hVOR) sind bei Beschwerden meist mit höhergradigen Auswirkungen verbunden als im Fall verdeckter Sakkaden.

Unabhängig vom Schaden finden sich graduell unterschiedliche vestibuläre Kompensationsmechanismen

Unabhängig vom Schaden finden sich graduell unterschiedliche vestibuläre Kompensationsmechanismen. Bei beidseitigen Störungen (z. B. hVOR) hingegen sind die Kompensationsmechanismen der Erfahrung nach deutlich geringer ausgeprägt als bei einseitigen Störungen [3, 4].

### Eigenschaften peripherer Vestibulopathien mit Anfallscharakter

Episodische periphere Vestibulopathien (z. B. M. Menière, BPLS) erfordern im Vergleich zu dauerhaften sensorischen Störungen eine andere Herangehensweise sowie krankheitsspezifische Bewertungskriterien (temporäres Auftreten, Besserungs- oder Verschlechterungstendenz im Zeitverlauf, ggf. Begleiterkrankungen wie Hörstörungen, Tinnitus und Komorbiditäten, wie eine vestibuläre Migräne oder ein funktioneller Schwindel). Im Vordergrund steht eine Bewertung der Anfallsschwere in Relation zur Anfallsdauer. Objektive Hinweise (z. B. Lagerungsnystagmus beim BPLS) sollten bei der Bewertung berücksichtigt werden [3, 4].

## Bewertungskriterien im Rechtskontext

### Periphere Vestibulopathien ohne Anfallscharakter

Dauerhafte Beeinträchtigungen mit „Schwindel“ wurden bisher entsprechend der Tabelle für „vestibuläre Störungen“ („Schwindeltabelle“) nach Stoll (1979, novelliert 2009) beurteilt [6, 7]. Die Bewertung erfolgt darin vordergründig über subjektive Eindrücke des zu Begutachtenden und subjektive Tests mithilfe der Parameter „Intensität“ (subjektive Beschwerden bei „Schwindel“) und „Belastung“ (Ergebnis vorwiegend subjektiver Testverfahren). In der aktuellen Fassung der Versorgungsmedizin-Verordnung findet sich eine modifizierte Tabelle für „Gleichgewichtsstörungen“ für den HNO-Fachbereich unter der Rubrik „Hör- und Gleichgewichtsorgan“ [20] im Abschnitt B 5.3 (Tabelle für „Gleichgewichtsstörungen“). Dass es sich im Vollbeweis um periphere Vestibulopathien (nicht um „Schwindel“, ohne Nachweis einer peripheren Vestibulopathie) handeln muss, wird meist nicht berücksichtigt.

Neue Tabellenvorschläge (Tabelle für *„periphere Vestibulopathien“*) ohne Anfallscharakter zur Einschätzung/Bewertung von MdE (Minderung der Erwerbsfähigkeit)/GdB (Grad der Behinderung) und GdS (Grad der Schädigungsfolgen) auf der Grundlage *objektiver* Methoden existieren seit 2023 [3–5].

### Periphere Vestibulopathien mit Anfallscharakter

In der aktuellen Versorgungsmedizin-Verordnung findet sich eine historische MdE/GdB/GdB-Tabelle für den „M. Menière“ [8] sowie eine modifizierte Fassung für die „Menière-Krankheit“ mit 3 Schweregraden und Verlaufsformen [19].

Einen aktualisierten Tabellenvorschlag auf der Basis aktueller wissenschaftlicher Erkenntnisse und mit objektiven Bewertungskriterien gibt es seit 2023 [4].

Die gutachterliche Bewertung des BPLS erfolgt seit 2012 mithilfe einer gesonderten MdE/GdB/GdS-Bewertungstabelle [1], dabei wird ein objektiver diagnostischer Nachweis einer peripheren Vestibulopathie (z. B. eines Nystagmus) gefordert. Die gutachterliche Bewertung (z. B. freies Intervall, Ausheilung) wurde kürzlich (2021) anhand neuer Aspekte der HNO-Lehrmeinung auf den Stand des Wissens aktualisiert [2].

### Beurteilung in ausgewählten Rechts- und Versicherungsbereichen

Mit der MdE werden die individuellen Einschränkungen auf dem allgemeinen Arbeitsmarkt eingeschätzt. Die Höhe der MdE (Angabe in %) reflektiert den Schweregrad der Folgen eines Unfalls oder einer Berufskrankheit. Bei peripheren Vestibulopathien muss ggf. eine berufliche Betroffenheit berücksichtigt werden.

Der Grad der Behinderung (GdB; dimensionslos, Staffelung in 10er-Schritten) beinhaltet final und der Grad der Schädigungsfolgen (GdS) kausal die körperlichen, seelischen, sozialen und geistigen Auswirkungen einer Gesundheitsstörung auf der Basis eines festgestellten (gesicherten) Gesundheitsschadens (z. B. periphere Vestibulopathie). Die in der aktuellen Fassung der Versorgungsmedizin-Verordnung (s. [7]) enthaltenen „Tabellen“ entsprechen Modifikationen der jeweils gültigen MdE/GdB/GdS-Tabellen. Gerade bei peripheren Vestibulopathien fehlt in der aktuellen Fassung ein eindeutiger Fokus auf „periphere Vestibulopathien“ sowie die Hinzuziehung objektiver Befunde. „Schwindel“ wird häufig auch dem HNO-Fachgebiet zugeordnet, wenn dieses als Begleitsymptom affektiver Störungen oder anderer fachfremder Erkrankungen auftritt.

Die HNO-Begutachtung bei „Schwindel“ orientiert sich an den unterschiedlichen Rechts- und Versicherungsgebieten

Im Rahmen der gesetzlichen Rentenversicherung (Frage der Erwerbsminderung) erfolgt die Feststellung eines positiven und negativen Leistungsbilds mit der Beurteilung von möglichen Tätigkeiten, ggf. mit individueller Einschränkung und zeitlicher Eingrenzung. Die Begutachtung orientiert sich an der Internationalen Klassifikation der Funktionsfähigkeit, Behinderung und Gesundheit (ICF, International Classification of Functioning, Disability and Health).

In der privaten Berufsunfähigkeitsversicherung wird anhand der Tätigkeitsbeschreibung und der festgestellten Gesundheitsstörung im HNO-Fachbereich (periphere Vestibulopathie) die Feststellung der Invalidität als abstrakter Körperschaden (Gliedertaxe) vorgenommen.

Für den Bereich der privaten Unfallversicherung sollte der Invaliditätsgrad für periphere Vestibulopathien analog nach den gültigen MdE/GdB/GdS-Tabellen eingeschätzt werden [21], da keine separaten Tabellen (Gliedertaxe) vorhanden sind.

Im Rahmen der Unfallfürsorgeversicherung für Beamte können die dienstunfallbedingten, unmittelbaren Unfallfolgen prozentual ebenfalls nach den gültigen Tabellenwerken eingeschätzt werden.

Für alle Rechts- und Versicherungsgebiete gilt, dass eine Gesundheitsstörung (periphere Vestibulopathie) im HNO-Fachbereich im Vollbeweis mithilfe objektiver Diagnostik gesichert sein muss. Das unspezifische, subjektive, fachübergreifende Symptom „Schwindel“ allein ist nicht ausreichend für eine Bewertung.

## Praktische Anwendung: Fallbeispiele

### Periphere Vestibulopathien

**Abb. 1:**
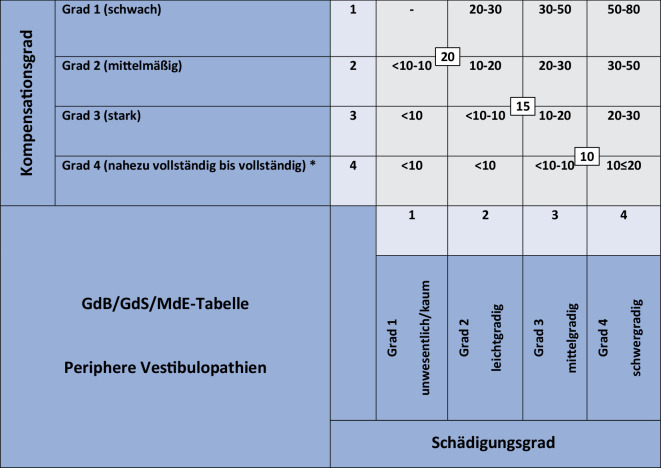
Neue MdE/GdB/GdS-Bewertungstabelle für ein- und beidseitige periphere vestibuläre Störungen. Ableitung von MdE/GdB/GdS aus dem ermittelten Organschaden und der vestibulären Kompensation. (*Asterisk* Bei einer vollständigen vestibulären Kompensation ohne Beschwerden keine MdE/GdB/GdS vorliegend). *GdB* Grad der Behinderung, *GdS *Grad der Schädigungsfolgen, *MdE *Minderung der Erwerbsfähigkeit

Der neue MdE/GdB/GdS-Tabellenvorschlag für periphere Vestibulopathien [4] bezieht sich auf alle ein- und beidseitigen sensorischen und/oder neuralen Gesundheitsstörungen im HNO-Fachbereich (periphere Vestibulopathien) mit dauerhaften Beschwerden. In Analogie zur Begutachtung von Hörstörungen [22] erfolgt die Bewertung über eine separate Einschätzung von „*Organschaden*“ und der *vestibulären Kompensation*. Mit den ermittelten Schweregraden kann in die MdE/GdB/GdS-Tabelle (Abb. 1) eingegangen werden [4].

#### Fallbeispiel 1: unilaterale Vestibulopathie. Frage der Höhe des GdB.

59-jährige Klägerin, Sozialgericht. 2021 akute unilaterale Vestibulopathie links. Strittige Frage der Höhe des GdB. Sitzende Tätigkeit (Sekretärin). Seit > 24 Monaten Beschwerden bei schnellen Bewegungen, „verschwimmende“ Bilder bei schnellen Kopfbewegungen. Intensive Physiotherapie über 1 Jahr, Kraft‑, Koordinations- und Balancetraining mit Besserungstendenz. Keine thermische Erregbarkeit links, hVOR-Gain links 0,1, offene Rückstellsakkaden links. Spontannystagmus (videookulographisch) etwa 5°/s. Verstärkung bei Provokation. Statische Prüfungen ohne bis schwache Normabweichungen, dynamische Prüfungen mit schwachen bis mittelmäßigen Normabweichungen. Organschadensgrad „schwergradig“ (Grad 4), „Vollschaden“ aller 5 Sensoren. Grad der vestibulären Kompensation mittelmäßig (Grad 2). GdB-Bewertung: Gesamt-GdB 30 (unilaterale Vestibulopathie ICD 10: H81.2 links) auf Zeit. Neubewertung nach 3 Jahren.

### Benigner paroxysmaler Lagerungsschwindel

**Abb. 2:**
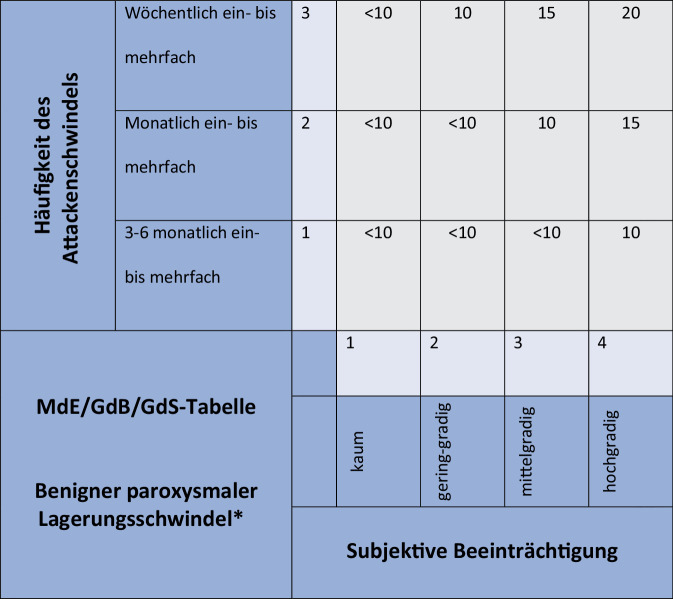
MdE/GdB/GdS-Tabelle für die Bewertung des benignen paroxysmalen Lagerungsschwindels (BPLS; [1]). (*Asterisk* Vorherige Objektivierung, z. B. Nystagmus und eine Einordnung nach den diagnostischen Kriterien [13] empfohlen). *GdB* Grad der Behinderung, *GdS *Grad der Schädigungsfolgen, *MdE *Minderung der Erwerbsfähigkeit

Bei der gutachterlichen MdE/GdB/GdS-Bewertung des gutartigen Lagerungsschwindels werden objektive Kriterien gefordert (Beobachtung und Dokumentation eines bogengangspezifischen Lagerungsnystagmus nach den aktuell gültigen diagnostischen Kriterien, Lehrmeinung, s. Bàràny-Konsensus-Dokumente; Abb. 2; [1, 13]).

Es empfiehlt sich beim BPLS eine Videodokumentation

Es empfiehlt sich eine Videodokumentation (Kamera oder Smartphone) oder ein objektive video- oder elektronystagmographische Analyse. Im Vordergrund stehen traumatische Ereignisse (Schädelprellung, ICD-10 S00), mit Kopfplatzwunde/Hämatom und/oder einer Fraktur (S02.1). Strukturelle Schäden können selten auch fehlen. Die Erkrankung tritt meist temporär auf, kann rezidivieren (etwa ein Drittel der Fälle), jedoch muss mittels objektiver Diagnostik auch nach weiteren sensorischen Schäden gesucht werden (ggf. integrative Beurteilung). Eine Ausheilung ist nach einem symptomfreien Intervall von 1 Jahr gegeben. Das freie Intervall kann bis zu 3 Monate betragen. Das belegen allgemeine Erfahrungswerte und die wissenschaftliche Literatur (Phase 1: Trauma: Lösen von Otokonien/Partikeln, Phase 2. Dislokation: eine kritische Masse von Partikeln wird in den Bogengang bewegt; Phase 3. Induktion der Erkrankung: Symptomatik eines BPLS durch spezifische Kopf-Körper-Bewegungen; s. [2]). In den meisten Fällen liegt eine zeitlich begrenzte Einschränkung vor. Die Prognose ist gut. Rezidive kommen jedoch vor.

#### Fallbeispiel 2: benigner paroxysmaler Lagerungsschwindel.

44-jährige Klägerin. Sozialgericht. Frage des Vorliegens eines BPLS und ggf. Höhe der MdE. Wegeunfall (Fahrradsturz) 2015 Sturz auf den Hinterkopf mit Kopfplatzwunde und weitere Körperverletzungen (Hand). Erstmaliges Auftreten und objektive Dokumentation eines gutartigen Lagerungsschwindels 36 Tage nach dem Unfall. Letzte Dokumentation 96 Tage nach dem Unfall. Keine Rezidive nach erfolgreicher Therapie. Danach kein BPLS mehr objektiv nachweisbar, aber „Schwindel“ (ICD 10 R42). Objektiv normale periphere vestibuläre Funktion. Neurologisch und neuropsychologisch normale Befunde. Keine funktionelle Störung. Arbeitsfähigkeit. Beurteilung: MdE auf Zeit (10 %) vom ersten Auftreten bis zur letzten Dokumentation des BPLS. Die Kopfplatzwunde als objektiver Nachweis spricht für ein adäquates Trauma und einen kausalen Zusammenhang. Diagnose: „Benigner paroxysmaler Lagerungsschwindel (H81.1) infolge Schädelprellung“ (ICD-10 S00.98) mit nachweisbaren strukturellen Schädigungen (Kopfplatzwunde, ICD-10 S01.0) für 60 Tage.

### M. Menière

Es sollte ein objektiver Nachweis nach den aktuellen Diagnosekriterien gefordert werden (z. B. periphere Vestibulopathie, Nystagmus, Hörstörung, Tinnitus) und ggf. eine Abgrenzung von der vestibulären Migräne (schwierig, ggf. als Komorbidität vorhanden, beim M. Menière meist ausgeprägte Hörstörung) erfolgt sein. Für die Diagnosestellung sind die aktuellen diagnostischen Kriterien [13] und ein hoher Wahrscheinlichkeitsgrad (gesicherte Diagnose) erforderlich. Die Diagnose eines „definitiven“ (engl. „definite“), klaren, feststehenden, M. Menière hat eine höhere diagnostische Wahrscheinlichkeit als die „wahrscheinliche Erkrankung“ („probable“).

Im neuen Tabellenvorschlag ist anstelle der früheren „Intensität“ die „Anfallsschwere“ getreten

**Abb. 3:**
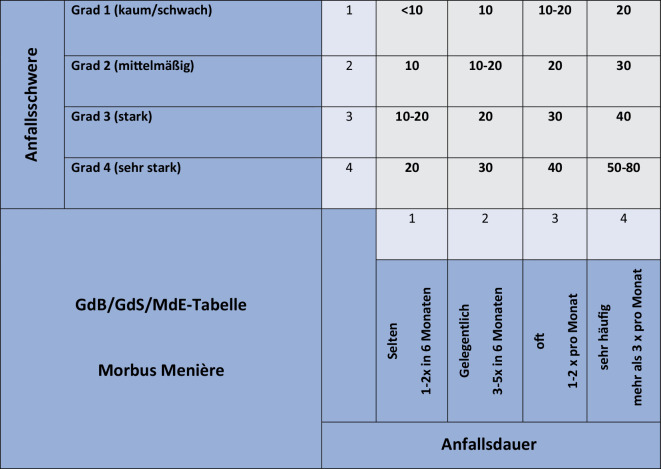
Neuer Vorschlag der MdE/GdB/GdS-Tabelle für den M. Menière. Sicherung der Erkrankung mit hoher Wahrscheinlichkeit erforderlich. *GdB* Grad der Behinderung, *GdS *Grad der Schädigungsfolgen, *MdE *Minderung der Erwerbsfähigkeit

Im neuen Tabellenvorschlag aus dem Jahr 2023 (Abb. 3; [4]) ist anstelle der früheren „Intensität“ die „Anfallsschwere“ getreten. Außerdem war eine zeitliche Anpassung an die aktuellen Diagnosekriterien erforderlich. Weiterhin erfolgte eine semantische Anpassung nach den Vorgaben mehrstufiger Rating-Skalen. Periphere Vestibulopathien, Hörstörungen, Tinnitus und funktionelle Störungen müssen separat bewertet werden [4].

#### Fallbeispiel 3: M. Menière.

48-jähriger Kläger (Sozialgericht). Streitige Frage der Höhe des Gesamt-GdB bei gesichertem M. Menière rechts (mit Tinnitus, Hörstörung, peripherer Vestibulopathie und funktionellem Schwindel). Die Anfälle treten dauerhaft (1- bis 2‑mal pro Monat) auf. Anfallsschwere im Mittel „stark“. Hierzu findet sich ein Ereignisprotokoll in den Akten. 2× stationärer Aufenthalt mit Nystagmus-Dokumentation. Tieffrequent und mittelfrequent stark akzentuierte Hörstörung rechts, links Normalgehör, plausibilisierbarer Tinnitus im Niederfrequenzbereich (500 Hz) rechts (Divergenztyp nach Feldmann) leicht über der Schwelle gelegen. Zusätzlich liegt eine periphere Vestibulopathie (Teilschaden: Untererregbarkeit etwa 75 % Seitendifferenz rechts in der thermischen Prüfung, Video-Kopfimpulstest regelrecht) vor. Der Kläger hat über Jahre Anfälle in einem Ereignisprotokoll dokumentiert und präsentiert einen Nystagmus auf der Handykamera, was auch in den Akten (Beweismittel) dokumentiert ist. Zusätzlich „ein Kommen und Gehen“ eines Schwindels mehrfach pro Monat (funktioneller Schwindel). Neurologie: Ausschluss einer vestibulären Migräne. Funktioneller Schwindel. Diagnose: Gesicherter M. Menière (ICD 10: H81.0, rechts), GdB-Bewertung für den M. Menière 30. Tinnitus rechts (H93.1; Einzel-GdB < 10), Hörstörung rechts (H91.3; Einzel-GdB 10), periphere Vestibulopathie rechts (Teilschaden; H83.2; kompensiert; Einzel GdB < 10) und funktioneller Schwindel (PPPD; F45.9; GdB 10) wurden separat bewertet. Integrative Bewertung: Gesamt-GdB 30. Neubewertung nach 3 Jahren.

## Fazit für die Praxis

- „Schwindel“ als subjektives Symptom lässt sich heute im HNO-Fachbereich mithilfe der objektiven Diagnostik aller 5 Sensoren im Rahmen einer gutachterlichen Fragestellung im HNO-Fachbereich differenzieren.
- Eine periphere Vestibulopathie kann so mit sehr hoher Wahrscheinlichkeit ausgeschlossen oder verifiziert werden.
- Episodische Schwindelsyndrome erfordern eine andere Herangehensweise als periphere Vestibulopathien mit dauerhaften Beschwerden.
- Die hier vorgestellten neuen Vorschläge zur Bewertung peripherer Vestibulopathien in Bezug auf Minderung der Erwerbsfähigkeit (MdE)/Grad der Behinderung (GdB) und Grad der Schädigungsfolgen (GdS) berücksichtigen objektive Kriterien, aktuelle Diagnosemethoden und wissenschaftliche Standards.
- Bei Diskrepanzen sollten mögliche Komorbiditäten (z. B. funktioneller Schwindel) geprüft und ggf. integrativ bewertet werden.
- Der gutachterlichen Freiheit wird bei der Diagnostik und der Bewertung Raum eingeräumt.
- Da sich die vestibuläre Kompensation, insbesondere bei einseitigen peripheren Vestibulopathien oder die Beschwerdesymptomatik (M. Menière), über die Zeit verändern kann, sollte die MdE/GdB/GdS bei der Erstbegutachtung zeitlich befristet ausgesprochen werden.
